# Do health professionals’ attitudes towards alcohol use matter for alcohol prevention efforts? Results from the WIRUS-OHS study

**DOI:** 10.1186/s12913-022-08400-9

**Published:** 2022-08-06

**Authors:** Tore Bonsaksen, Mikkel Magnus Thørrisen, Neda Hashemi, David Gimeno Ruiz de Porras, Randi Wågø Aas

**Affiliations:** 1 Department of Health and Nursing Sciences, Faculty of Social and Health Sciences, Inland Norway University of Applied Sciences, Elverum, Norway; 2 Department of Health, Faculty of Health Studies, VID Specialized University, Stavanger, Norway; 3 Department of Occupational Therapy, Prosthetics and Orthotics, Faculty of Health Sciences, Oslo Metropolitan University, Oslo, Norway; 4 Department of Public Health, Faculty of Health Sciences, University of Stavanger, Stavanger, Norway; 5 Southwest Center for Occupational and Environmental Health, Department of Epidemiology, Human Genetics, and Environmental Sciences, School of Public Health in San Antonio, The University of Texas Health Science Center at Houston, Houston, TX 77229 USA; 6 Center for Research in Occupational Health (CiSAL), Universitat Pompeu Fabra, 08002 Barcelona, Spain; 7 CIBER of Epidemiology and Public Health, 28029 Madrid, Spain

**Keywords:** Alcohol, Attitudes, Drinking, Norms, Occupational health services, Prevention

## Abstract

**Background:**

Use of alcohol is a major public health issue, representing the 7^th^ largest burden of disease in the world. Workplaces offer a unique arena for health initiatives addressing alcohol use, where occupational health services (OHS) personnel play an important role. However, we do not know if the extent of such initiatives may be influenced by personal drinking attitudes among OHS personnel. Thus, the aim of the study was to explore how drinking attitudes among OHS personnel were associated with their frequency of working with alcohol-related cases and with their views on alcohol prevention activities in the OHS.

**Methods:**

The WIRUS project included a cross-sectional survey of attitudes and practices among OHS personnel (*n* = 325) employed by Norwegian OHS services (*n* = 69), who informed about sociodemographic and professional characteristics, drinking attitudes, frequency of cases with alcohol-related issues, and perceptions toward the role of the OHS in primary, secondary, and tertiary alcohol prevention activities. Measures of associations were examined with linear and logistic regression models.

**Results:**

Drinking attitudes were unrelated to the frequency of working with alcohol-related cases. Physicians, psychologists, and nurses had higher frequency of working with alcohol-related cases, compared to those with other professional backgrounds (β = 0.46, *p* = 0.01). Drinking attitudes were also unrelated to attitudes towards primary/secondary/tertiary alcohol prevention activities in the OHS, while female OHS personnel were more positive towards increased primary alcohol prevention activities in the OHS (OR: 1.82, *p* < 0.05). Only marginal portions (1%-3%) of the variance in attitudes towards alcohol prevention activities in the OHS were accounted for by the models.

**Conclusion:**

This study did not find evidence of associations between OHS personnel’s drinking attitudes and their practices and attitudes towards alcohol prevention activities. The lack of association between OHS personnel’s attitudes towards alcohol use and their attitudes and practices relating to alcohol prevention in the workplace might point towards professionalism, as personal attitudes appear not to interfere with their priorities and professional mission. Given the small amount of outcome variance explained by the tested models, other variables should be used in future studies.

## Background

Regular alcohol use is reported by over 80% of the Norwegian adult population, with over two thirds reporting drinking every week [1, 2]. Since alcohol use and abuse has been linked with a wide range of disease and injury conditions [3, 4], and work-related effects such as increased sick leave [5–7] and reduced work productivity [8], the levels of regular drinking in Norway should clearly be considered a major public health priority.

A variety of alcohol use prevention strategies, mostly related to alcohol education, are put in place by national and local governments. These actions seem to have little to no evidence of producing any positive change [9] and may fail in engaging individuals [10]. Given most adults spent longer hours at work and that workplaces must provide a safe work environment, the workplace offers a unique opportunity for public health action [11–13]. For instance, research shows that up to 30% of employees may benefit from alcohol prevention interventions [14–17].

While occupational health services (OHS) are well positioned to provide alcohol-related interventions at the workplace, research on alcohol prevention efforts led by OHS personnel is limited [18–20]. Moreover, our earlier study in Norway found that alcohol prevention activity differed between the professional groups involved in implementing the prevention, and that perceived implementation barriers were significantly associated with lower prevention activity, as reported by OHS personnel [21]. Implementation barriers *internal* to the OHS’ organization (e.g., lack of time, resources, and competence) were associated with lower prevention activity, while barriers *external* to the OHS’ organization (barriers concerning employers and employees) were not. This pattern, which was evident for primary, secondary, and tertiary prevention activities, might indicate knowledge gaps in how OHS personnel perceive their own alcohol preventive effort. Notably, a majority of the OHS personnel agreed that employees’ alcohol consumption constitute a public health challenge, and that OHS should focus more on alcohol prevention targeting employees.

However, drinking attitudes among OHS personnel might interfere with or strengthen the role of OHS in prevention activities, and drinking norms and drinking culture aggregated on group and organizational levels might influence whether and how individual attitudes towards drinking translate into alcohol prevention practices. According to the theory of planned behavior [22], positive attitudes, in addition to perceived social norms and perceived control over the behavior, increase the likelihood of performing a given behavior. The theory applies to alcohol use given the longstanding empirical evidence for associations between positive attitudes towards drinking and higher alcohol consumption [23–26]. For instance, positive drinking attitudes among a group of Norwegian employees have been related to alcohol-related problems [27] such as employees with positive attitudes towards drinking were almost three times as likely to report alcohol problems than employees with more negative attitudes. The association was stronger among women than among men.

This study draws on the theory of planned behavior and findings substantiating relationships between alcohol-related attitudes and behaviors, while the focus is transferred onto OHS personnel. In line with that theory [22], a review indicated that health professionals’ alcohol prevention practice with clients was associated with their personal attitudes towards drinking [28]. However, the number of studies reviewed was small (*n* = 6) and each study was largely confined to one specific professional group (physicians [*n* = 2], nurses [*n* = 2], dentists [*n* = 1], mixed sample of health professionals [*n* = 1]), thus indicating a need for more research. The current study, the Norwegian national WIRUS-project (Workplace Interventions preventing Risky Use of alcohol and Sick leave) included a large survey about alcohol use [27, 29, 30], absenteeism and presenteeism [31], work environment perceptions [32, 33], and alcohol-related culture [34] among employees in private and public sector enterprises. Employees reporting risky use of alcohol (*n* = 800, 11.5% of all participants with valid scores on the screening instrument) were offered the option of being randomly assigned to a web-based intervention, a face to face intervention, or a control group, and 38% agreed to be randomized [35]. The WIRUS-project also included a survey about attitudes and practices among OHS personnel, who are often in the frontline when alcohol prevention is introduced in the workplace. The aims of the current study were to explore how drinking attitudes among OHS personnel were associated with their view of the OHS role and responsibility in alcohol prevention activities, particularly regarding their frequency of working with alcohol-related cases and whether the association between drinking attitudes and frequency of taking on alcohol-related cases was moderated by professional background, size of work unit, and work location. We also explored how drinking attitudes among OHS personnel were associated with their perceptions toward the role of the OHS in primary, secondary, and tertiary alcohol prevention activities.

## Methods

### Design

The WIRUS project included a cross-sectional survey of attitudes and practices among 357 OHS personnel employed by 69 OHS services in Norway. The current study used data from the WIRUS-OHS survey, which was conducted in 2018.

### Sample

In this study, 32 (9%) of the 357 OHS personnel were excluded from the analysis due to missing responses, rendering 325 OHS personnel to be included. The sample characteristics are shown in Table 1.

**Table 1:** Sample characteristics by type of drinking attitudes

Variables	Total	Liberal	Restrictive	*p-value*^*a*^
**Age** (M [SD])	48.9 (10.1)	47.9 (9.8)	50.0 (10.2)	0.06
**Gender**	(*n* [%])	(*n* [%])	(*n* [%])	0.28
Male	67 (20.6)	39 (58.2)	28 (41.8)	
Female	258 (79.4)	131 (50.8)	127 (49.2)	
**Experience from OHS work** (M [SD])	12.0 (9.1)	11.4 (9.0)	12.6 (9.1)	0.25
**Professional group**	(*n* [%])	(*n* [%])	(*n* [%])	0.62
Physician	42 (12.9)	22 (52.4)	20 (47.6)	
Psychologist	7 (2.2)	5 (71.4)	2 (28.6)	
Nurse	123 (37.8)	59 (48.0)	64 (52.0)	
Occupational therapist	10 (3.1)	7 (70.0)	3 (30.0)	
Occupational hygienist	27 (8.3)	12 (44.4)	15 (55.6)	
Nutritionist	1 (0.3)	1 (100.0)	0 (0.0)	
Physiotherapist	57 (17.5)	32 (56.1)	25 (43.9)	
Other^b^	58 (17.8)	32 (55.2)	26 (44.8)	
**Frequency of alcohol-related cases**		*n* (%)	*n* (%)	0.15
Never	58 (17.8)	34 (58.6)	24 (41.4)	
Less often than yearly	61 (18.8)	27 (44.3)	34 (55.7)	
Yearly	41 (12.6)	22 (53.7)	19 (46.3)	
Less often than monthly	73 (22.5)	46 (63.0)	27 (37.0)	
Monthly	66 (20.3)	30 (45.5)	36 (54.5)	
Weekly	26 (8.0)	11 (42.3)	15 (57.7)	
Daily	0 (0.0)	0 (0.0)	0 (0.0)	
**Attitudes towards OHS engagement with primary prevention**		*n* (%)	*n* (%)	0.30
Less than, or to the same extent as today	190 (58.5)	102 (54.3)	86 (45.7)	
More than today	135 (41.5)	66 (48.9)	69 (51.1)	
**Attitudes towards OHS engagement with secondary prevention**		*n* (%)	*n* (%)	0.67
Less than, or to the same extent as today	115 (35.4)	60 (53.1)	53 (46.9)	
More than today	210 (64.6)	108 (51.4)	102 (48.6)	
**Attitudes towards OHS engagement with tertiary prevention**		*n* (%)	*n* (%)	0.24
Less than, or to the same extent as today	187 (57.5)	99 (54.1)	84 (45.9)	
More than today	138 (42.5)	67 (48.6)	71 (51.4)	

^a^ Statistical tests are Chi Square (categorical variables) and independent t-tests (continuous variables) of the differences between liberal and restrictive groups

^b^The ‘other’ category consisted of e.g., medical secretaries, engineers, educationalists/teachers, economists, and social scientists

### Measures

#### Dependent variables

Frequency of alcohol-related casework was measured by responses to the question: “How often do you work with cases related to alcohol use (at the individual or group level)?” Response options were never (1), less often than yearly (2), yearly (3), less often than monthly (4), monthly (5), weekly (6), daily (7).

Attitudes towards working with alcohol use among non-risk employees (primary prevention), employees at risk of alcohol-related problems (secondary prevention), and employees with alcohol problems (tertiary prevention) were assessed with the question: “To what extent do you feel that your OHS should work with alcohol-related issues among employees in the following categories?” Categories listed were “employees with no known alcohol risk” (corresponding to primary prevention), “employees presumably drinking more than recommended” (corresponding to secondary prevention), and “employees with known alcohol problem” (corresponding to tertiary prevention). In relation to each category, the participants were asked to indicate whether they felt the OHS should work with the group “less than today” (1), “to the same extent as today” (2) or “more than today” (3). Due to few responses indicating “less than today”, these variables were all recoded to indicate “less than, or to the same extent as today” (0), or “more than today” (1).

#### Independent variables

Drinking attitudes were measured with the Drinking Norms Scale (DNS) [36], using a version translated into Norwegian by the research team following standard guidelines for translating questionnaires [37]. The DNS is a 7-item scale focused on attitudes toward drinking in general (three items) and work-related drinking (four items). Responses were coded on a 4-point Likert scale (1 = strongly disagree; 2 = disagree; 3 = agree; 4 = strongly agree). To compute the DNS summary scale, negatively worded items (i.e., items 6 and 7) were reverse scored, and a mean score for all seven items was calculated so that higher scores indicated more liberal drinking attitudes. Previous research has shown that the scale items relate to a single underlying dimension and that the internal consistency between items is good (Cronbach’s *α* = 0.79) [36]. Acceptable internal consistency (Cronbach’s *α* = 0.71) was also shown for the Norwegian translation of the DNS tool when used in a sample of Norwegian employees [27]. In this study of OHS personnel, the internal consistency of the seven DNS items was not as good as desirable (Cronbach’s *α* = 0.63; mean inter-item correlation = 0.20). The median split value was used to compare OHS personnel with liberal (*Md* ≥ 2) versus restrictive (*Md* < 2) drinking attitudes.

#### Covariates

Potential confounders were age (in years), gender (male = 1, female = 2), years worked in an OHS, and professional background (occupational therapist, nutritionist, physiotherapist, physician, psychologist, nurse, occupational hygienist, or other). In view of previous research demonstrating a higher frequency of working with alcohol-related cases among physicians, psychologists, and nurses, compared to OHS personnel with other professional backgrounds [21], professional background was dichotomized for the inferential analyses (physician, psychologist, or nurse = 1, occupational therapist, nutritionist, occupational hygienist, physiotherapist, or other = 0). For the interaction analyses, we also included size of work unit, using the median split to distinguish small units (1–7 employees) from larger units (8 or more employees), and work location, distinguishing between OHS units located in the Northern, Southern, Eastern, Western, and Middle regions of Norway.

### Statistical analysis

Descriptive analyses were performed for all variables, and group comparisons (Chi-square tests and independent *t*-tests) were made for OHS personnel with liberal versus restrictive drinking norms. Associations between OHS personnel’s drinking norms and their frequency of having an alcohol-related caseload were analyzed with single and multiple linear regression analysis. The linear regression analyses included age, gender, professional background (dichotomized), years of OHS experience, and three interaction terms (DNS × professional background; DNS × size of OHS unit; DNS × location of OHS unit) as covariates. In order to include possible predictors in a multiple model while also minimizing the risk of losing statistical power, variables associated with the outcome with *p* < 0.20 when used as single predictor were carried over to the multiple linear regression analysis [38]. Single and multiple binary logistic regression analyses were used to examine associations between OHS personnel’s drinking attitudes and their attitudes towards the OHS personnel’s role in primary, secondary, and tertiary prevention of alcohol problems. Variables associated with the outcome with *p* < 0.20 when used as single predictor were carried over to the multiple logistic regression analysis.

## Results

### Drinking attitudes

Among the OHS personnel, 155 (47.7%) were classified as ‘restrictive’ based on their scores on the DNS (score below 2, the median sample score), while 170 (52.3%) were classified as ‘liberal (score at or above 2). There were no statistically significant differences between liberal and restrictive OHS personnel on any of the measures.

### Frequency of working with alcohol-related cases

Results between sample characteristics and frequency of working with alcohol-related cases are shown in Table 2. The unadjusted analyses showed that the frequency was higher among OHS personnel who were older, had more OHS experience, and had professional background as physician, psychologist, or nurse. The association between the DNS scores, whether used as a continuous or categorical variable, and the outcomes was not statistically significant. Professional group interacted with DNS (continuous) scores in predicting frequency of alcohol-related caseload, while there was no interaction between size of work unit, or location of work unit, and the DNS score.

**Table 2:** Relationships between sample characteristics and frequency of working with alcohol-related cases

Characteristics	Unadjusted			Adjusted		
	B^a^ (95% CI)^b^	β^c^	*p*-value	B^a^ (95% CI)^b^	β^c^	*p*-value
One-year increase in age	0.04 (0.02–0.05)	0.24	< 0.001	0.02 (-0.00–0.04)	0.12	0.07
Female gender	-0.04 (-0.47–0.39)	-0.01	0.85	-	-	-
One-year increase in OHS experience	0.03 (0.01–0.05)	0.19	0.001	0.01 (-0.01–0.03)	0.06	0.35
Professional group^d^	1.41 (1.09–1.72)	0.44	< 0.001	1.46 (0.36–2.56)	0.46	0.01
One-point increase in DNS score	-0.15 (-0.59–0.29)	-0.04	0.51	-	-	-
DNS × professional group	0.67 (0.51–0.83)	0.41	< 0.001	-0.08 (-0.64–0.47)	-0.05	0.77
DNS × size of work unit	-0.09 (-0.27–0.10)	-0.05	0.36	-	-	-
DNS × location of work unit	-0.00 (-0.07–0.06)	-0.01	0.91	-	-	-
Explained variance (*R*^2^)					21.7%	< 0.001

^a^ Beta coefficient from linear regression

^b^ 95% confidence intervals

^c^ standardized beta coefficient

^d^ the reference category includes occupational therapist, occupational hygienist, nutritionist, physiotherapist, and ‘other’, while the second category includes physician, psychologist, and nurse

The adjusted analysis revealed that the group of physicians, psychologists, and nurses had higher frequency of working with alcohol-related cases, compared to those with other professional backgrounds (β = 0.46, *p* = 0.01). Age, years of OHS experience, and the interaction between DNS score and professional group, were no longer statistically significant. When removing the interaction term from the multiple model, coefficients for the remaining covariates were practically unchanged (professional background: β = 0.41, *p* < 0.001).

### OHS personnel’s attitudes towards alcohol prevention activities in the OHS

Results between sample characteristics and the OHS personnel’s attitudes towards alcohol prevention activities in the OHS are shown in Table 3. DNS scores were unrelated to attitudes towards alcohol prevention activities in the OHS across all three levels of prevention (primary, secondary, and tertiary) both for the continuous and dichotomized versions of the DNS scale. Female OHS personnel were more positive towards increased primary alcohol prevention activities in OHS, compared to males (OR: 1.82, *p* < 0.05). No variables were associated with attitudes towards secondary or tertiary alcohol prevention activities in the OHS.

**Table 3:** Relationships between sample characteristics and attitudes towards increased alcohol prevention activities in the OHS

Characteristic	Primary prevention		Secondary prevention		Tertiary prevention	
	Unadjusted	Adjusted	Unadjusted	Adjusted	Unadjusted	Adjusted
	OR^a^ (95%CI)^b^	OR^a^ (95%CI)^b^	OR^a^ (95%CI)^b^	OR^a^ (95%CI)^b^	OR^a^ (95%CI)^b^	OR^a^ (95%CI)^b^
One-year increase in age	1.00 (0.98–1.02)	-	1.00 (0.97–1.02)	-	0.99 (0.97–1.01)	-
Female gender	1.73 (0.98–3.07)	1.82* (1.01–3.28)	1.11 (0.64–1.94)	-	1.12 (0.65–1.93)	-
One-year increase in OHS experience	1.02 (0.99–1.04)	1.02 (1.00–1.05)	1.00 (0.97–1.02)	-	0.98 (0.96–1.01)	-
Professional group^c^	1.40 (0.90–2.18)	1.29 (0.82–2.03)	1.45 (0.92–2.29)	1.45 (0.92–2.29)	1.05 (0.68–1.63)	-
One-point increase in DNS score	0.72 (0.41–1.25)	-	0.72 (0.40–1.28)	-	0.62 (0.35–1.08)	0.62 (0.35–1.08)
Nagelkerke R^2^		3.3%*		1.1%		1.2%

^a^ Odds ratio from logistic regression

^b^ 95% confidence intervals

^c^ the reference category includes occupational therapist, occupational hygienist, nutritionist, physiotherapist, and ‘other’, while the second category includes physician, psychologist, and nurse

^*^p < 0.05

## Discussion

Individual attitudes towards drinking did not contribute to explain the OHS personnel’s frequency of working with alcohol-related cases in their practice, nor did it contribute to explain their attitudes towards increasing the emphasis on alcohol prevention in the OHS where they worked. An alcohol-related caseload was more often found among physicians, psychologists, and nurses, compared to employees with other professional background, and adjusting for all variables, the link between professional background and frequency of alcohol-related caseload was independent from drinking attitudes. Female OHS personnel were more likely than males to support the idea of increasing primary alcohol prevention activities where they worked.

Attitudes are often emphasized as a key component when attempting to predict behavior and constitute a primary element in the theory of planned behavior for explaining the formation of behavioral intentions [22]. Crano and Prislin [39] (p. 360) stated that “[b]ecause attitudes predict behavior, they are considered the crown jewel of social psychology”. However, the predictive power of attitudes has been questioned. Already in the 1960s, Wicker [40] concluded that attitudes rarely account for more than nine percent of the variability in behavior. More recently, the relationship between attitudes and behavior has been portrayed as a complex association that is determined and affected by aspects of both the attitudes and the behaviors in question. For instance, readily accessible and strongly held attitudes are far more predictive than weak attitudes that are rarely activated [41, 42]. Moreover, a variety of extraneous factors may either promote or disrupt the degree to which behavior is accounted for by attitudes [43, 44]. Situational, contextual and sociocultural factors may cause people to act inconsistently with their attitudes [45].

In the current study, while the OHS personnel reported relatively restrictive attitudes, variations in attitudes were not related to differences in behaviors. We should note that the behaviors in question were professional behaviors targeting others, specifically taking on an alcohol-related caseload and conducting alcohol prevention activities among their clientele. Thus, restrictive attitudes towards alcohol use held among OHS personnel do not imply a greater inclination to become involved in alcohol prevention activities as part of their professional role. This may well be interpreted as professionalism, in this case by not allowing personal attitudes to get in the middle of their professional priorities and mission. It is also possible that attitudes concerned with respect for privacy and personal lifestyle more strongly influence the degree to which OHS personnel become involved in alcohol prevention. In line with this view, a previous study from the WIRUS-project showed that considering alcohol use to be a private matter was reported as the most salient barrier for OHS personnel’s involvement in alcohol prevention [21].

The workplace has been emphasized as a priority setting for health promotion and illness prevention [46], indicating that OHS personnel are well positioned to have a substantial impact on public health. However, their work is influenced by sociocultural and subjective norms in the relevant OHS that promote some actions and discourage others. Therefore, sociocultural norms may disrupt the association between personally held attitudes and behavioral outcomes among them. As such, the occupational health setting may constitute a situation in which behavior is more a function of situational demands and norms than a function of idiosyncratic factors, such as personally held attitudes [47]. The personal attitudes among OHS personnel regarding alcohol consumption may thus not translate into how often they actually work with alcohol-related cases or their perceptions of how often their OHS unit should conduct such preventive efforts. Correspondingly, an earlier study of alcohol prevention activity in Norwegian OHS units concluded that situational factors such as time, resources and training constituted the primary predictors for implementing alcohol prevention interventions [21].

The inclination to take on alcohol-related cases may in part be an effect of perceived knowledge and competence. Perceived lack of competence has been shown to be a significant barrier for alcohol prevention activities across all levels of prevention [21], and possibly, perceived competence may be linked with professional background. A tentative causal chain from education, through perceived competence, to behavior would contribute to explain the association between professional background and frequency of taking on alcohol-related cases. In Norway, education programs in medicine and psychology have a duration of six years, which is double the study time compared to, for instance, occupational therapists and physiotherapists. By default, different education programs also lead to different sets of knowledge and skills. The content of the study programs in medicine and psychology are likely to be more attuned to health problems involving alcohol use, compared to study programs such as occupational therapy and physiotherapy. One might therefore expect physicians and psychologists to have higher levels of perceived competence, and consequently to be more prone to include alcohol-related cases in their work schedule. Alternatively, or in addition, the process of allocating certain groups of personnel to work with alcohol-related cases may also be a function of the workplace organization and culture. Differences between professional groups may therefore also be explained by workplace organization and culture, as opposed to OHS personnel’s individual choice based on perceived competence.

Nursing education programs, however, have three years duration, similar to education programs in occupational therapy and physiotherapy. Therefore, higher perceived competence due to a more comprehensive education program cannot explain why nurses appear to be more involved in alcohol prevention, compared to OHS employees with other professional background. However, by their numbers alone, the nursing profession holds a strong position on most healthcare arenas, and in Norway, nurses have a well-developed system for supervision, further education, and career development in the workplace. Training and support in the specific work setting has been found to be related to higher levels of role adequacy, role legitimacy, motivation, and role satisfaction [48]. In Norway, nurses have also been found to become less psychologically distressed during the first three years after graduation [49], pointing towards a gratifying work situation. Possibly, the training and support structures available within the nursing profession may have contributed to their higher level of involvement in taking on alcohol-related cases, compared to employees who have completed different, yet equally comprehensive, education programs. These are, however, a few possible interpretations and should not be considered comprehensive or definitive explanations for the detected differences between the professional groups.

After adjustment for OHS experience and professional background, OHS personnel who were women were more positive than men towards increasing primary prevention activities targeting alcohol use. While the proportion of men with liberal drinking attitudes was somewhat larger than the corresponding proportion of women, this difference was not statistically significant, nor was the association between drinking attitudes and attitudes towards increased alcohol prevention activities in the OHS. Therefore, rather than being due to differences in drinking attitudes, it is more likely that OHS personnel who are men are more inclined to consider alcohol use a matter of personal lifestyle and choice and that privacy concerning alcohol use should be respected. Conversely, OHS personnel who are women may be more open to address alcohol use as a health-related issue with no other pretext than general knowledge about alcohol use and its harmful consequences. In support of this reasoning, previous research has found women to have more faith than men in the effectiveness of prevention and treatment efforts concerned with the use of alcohol and other substances [50]. However, these are speculations that will need to be addressed properly in future studies.

### Study strengths and limitations

One earlier review included studies where the sample in each original study was primarily limited to one specific professional group [28]. In comparison, a strength of our study is the use of a more heterogeneous sample, consisting of OHS personnel representing several professional groups practicing in the OHS setting. The heterogeneous sample increases the representativity of the study findings across professional groups working in the occupational health services. However, our study is limited due to a small sample size, leading to low statistical power and consequently to possible issues concerning Type II-error. Therefore, the small sample size detracts from our ability to generalize the study results to the larger population of OHS personnel and may have caused effects in the data (group differences and associations between variables) to go unnoticed. Generalizability is also reduced due to the recruitment procedure. The recruitment of the sample allowed for possible selection bias, and we do not know to what extent the sample characteristics correspond with the larger population of OHS personnel.

All data were self-reported, and while this strategy is required for obtaining data on attitudes, it also renders the possibility that data are influenced by social desirability motives among the participants. Hence, the self-reported data collected in the study may be skewed by such bias. We did not collect data about the participants’ own use of alcohol. Higher alcohol consumption has been found to be related to more liberal attitudes towards alcohol use as measured with the DNS [27], and the level of alcohol consumption among OHS personnel might also be related to their inclination to engage in alcohol prevention activities as part of their practice. Future studies may therefore include alcohol use as an additional possible predictor of OHS personnel’s alcohol prevention practices.

Moreover, a positive correlation between higher alcohol use and higher DNS scores might be indicative of DNS validity for the target group. While the original DNS scale has shown good validity and reliability [36], the Norwegian translation of the scale has not yet been psychometrically investigated. Thus, information about various aspects of its validity (content, concept, convergent, and discriminant validity) in Norwegian contexts is lacking. In contrast to the findings in a previous study performed with a large and heterogeneous sample of employees in the Norwegian workforce [27], the DNS items had lower than desirable internal consistency in our study. Thus, future research will need to examine the measurement properties of the Norwegian translation of the scale. The dependent variables used in the study were self-developed, and also with measurement properties unknown.

## Conclusion

In this study, drinking attitudes among OHS personnel were examined in relationship to frequency of working with alcohol-related cases and attitudes toward the role of the OHS in alcohol prevention activities. We found no evidence of individual drinking attitudes being related to OHS personnel’s attitudes or practice related to alcohol prevention activities. Group differences in the inclination to take on alcohol-related cases may point towards higher competence and well-functioning support systems within the professions as possible working mechanisms. Differences in alcohol-related prevention practices between professional groups were independent of drinking attitudes. The models assessed in the study explained only marginal portions of the outcome variance, suggesting that other variables should be used in the pursuit of a good model for explaining alcohol-related practice among OHS personnel.

## Data Availability

Data from the WIRUS OHS study are available from the project owner (University of Stavanger, Faculty of Health Sciences, Department of Public Health, Research group Societal Participation in School and Work) by principal investigator and project manager Randi Wågø Aas on reasonable request.

## Abbreviations

B: Beta coefficient
β: Standardized beta coefficient
CI: Confidence Interval
DNS: Drinking Norms Scale
M: Mean
n: Sample size
OHS: Occupational Health Services
OR: Odds Ratio
p: Probability value
SD: Standard Deviation
WIRUS: Workplace Interventions preventing Risky Use of alcohol and Sick leave
