# Correction: Do health professionals’ attitudes towards alcohol use matter for alcohol prevention efforts? Results from the WIRUS-OHS study

**DOI:** 10.1186/s12913-022-08533-x

**Published:** 2022-09-14

**Authors:** Tore Bonsaksen, Mikkel Magnus Thørrisen, Neda Hashemi, David Gimeno Ruiz de Porras, Randi Wågø Aas

**Affiliations:** 1 Department of Health and Nursing Sciences, Faculty of Social and Health Sciences, Inland Norway University of Applied Sciences, Elverum, Norway; 2 Department of Health, Faculty of Health Studies, VID Specialized University, Stavanger, Norway; 3 Department of Occupational Therapy, Prosthetics and Orthotics, Faculty of Health Sciences, Oslo Metropolitan University, Oslo, Norway; 4 Department of Public Health, Faculty of Health Sciences, University of Stavanger, Stavanger, Norway; 5 Southwest Center for Occupational and Environmental Health, Department of Epidemiology, Human Genetics, and Environmental Sciences, School of Public Health in San Antonio, The University of Texas Health Science Center at Houston, San Antonio, TX 77229 USA; 6 Center for Research in Occupational Health (CiSAL), Universitat Pompeu Fabra, 08002 Barcelona, Spain; 7 CIBER of Epidemiology and Public Health, 28029 Madrid, Spain

**Correction: BMC Health Serv Res 22, 1004 (2022)**

**https://doi.org/10.1186/s12913-022-08400-9**

Following publication of the original article [1], the authors reported missing information in the ‘Funding’ section.

The statement in the ‘Funding’ section originally read: This study is funded by the Norwegian Directorate of Health and the Research Council of Norway. The funding bodies had no role in the design of the study, nor in data collection, analysis, or data interpretation.

The statement in the ‘Funding’ section should read: This study is funded by the Norwegian Directorate of Health and the Research Council of Norway. The funding bodies had no role in the design of the study, nor in data collection, analysis, or data interpretation.

DGRdP was partially funded by the Southwest Center for Occupational and Environmental Health (SWCOEH), a National Institute for Occupational Safety and Health (NIOSH) Education and Research Center at The University of Texas Health Science Center at Houston School of Public Health, and awardee of Grant No. 5T42OH008421 from the (NIOSH)/Centers for Disease Control and Prevention.

The original article [1] has been updated.
